# Comparative Evaluation of ECG and Motion Signals in the Context of Activity Recognition and Human Identification

**DOI:** 10.3390/s25196040

**Published:** 2025-10-01

**Authors:** Ludwin Molina Arias, Magdalena Smoleń

**Affiliations:** Department of Biocybernetics and Biomedical Engineering, AGH University of Krakow, 30 Mickiewicza Avenue, 30-059 Kraków, Poland; arias@agh.edu.pl

**Keywords:** cluster analysis, human activity recognition, multimodal biometric recognition, unsupervised learning, signal processing

## Abstract

This study presents a comparative analysis of electrocardiogram (ECG) and accelerometer (ACC) data in the context of unsupervised human activity recognition and subject identification. Recordings were obtained from 30 participants performing activities of daily living such as walking, sitting, lying, cleaning the floor, and climbing stairs. Distance-based signal comparison methods and clustering techniques were employed to evaluate the feasibility of each modality, both individually and in combination, to discriminate between individuals and activities. Results indicate that ACC signals provide superior performance in activity recognition (NMI = 0.728, accuracy = 0.817), while ECG signals show higher discriminative power for subject identification (NMI = 0.641, accuracy = 0.500). In contrast, combining ACC and ECG signals yielded lower scores in both tasks, suggesting that multimodal fusion introduced additional variability. These findings highlight the importance of selecting the most appropriate modality depending on the recognition objective and emphasize the challenges associated with multimodal approaches in unsupervised scenarios.

## 1. Introduction

Multimodal sensing technologies are becoming increasingly important in applications such as health monitoring, smart environments, and biometric systems due to their ability to provide continuous and non-invasive observations [1,2]. Furthermore, recent work has explored how energy-autonomous wearable systems, powered by multimodal energy-harvesting technologies, can support long-term operation in such contexts [3]. Among the various biological signals, electrocardiogram (ECG) and accelerometer (ACC) data are two widely used modalities that contain physiological and behavioral information, respectively [4].

ECG signals are electrical recordings of cardiac activity, typically acquired through electrodes placed on the skin. These signals capture different physiological patterns and have been widely investigated for biometric identification [5,6,7,8,9,10], as well as for human activity recognition (HAR) based on cardiac dynamics [11,12,13]. In comparison, ACC signals, obtained from inertial measurement units (IMUs), encode the motion of body segments in a 3D space and are commonly used in HAR [14,15,16,17,18]. Moreover, ACC data have shown potential in identifying individuals based on behavioral characteristics such as gait patterns [19,20,21].

Although both modalities have been applied independently in recognition tasks, each has limitations. ECG signals can be sensitive to variations in emotional or physiological state and environmental noise [22], while ACC signals may lack the distinctiveness required for robust identity recognition. To mitigate these drawbacks, recent studies have proposed multimodal approaches that combine ECG and ACC data, utilizing the complementary strengths of both sources to improve recognition performance in classification of activities of daily living [23,24,25] and biometric identification [26,27,28]. A broader overview of multimodal sensing strategies for wearable health monitoring, including sensor fusion and data integration, is provided in [29].

Recognition tasks involving physiological and motion signals generally fall within the domain of machine learning, where supervised and unsupervised methods are commonly employed [30]. Supervised learning, which relies on labeled data to train models for classification or regression, has been the dominant approach due to its high performance and ability to achieve specific recognition objectives [31]. However, its applicability is often constrained by the need for large annotated datasets, which can be expensive, labor-intensive, and difficult to generalize across subjects and environments. In contrast, although less explored, unsupervised learning seeks to discover hidden structures in data without the need for labels, making it more adaptable to real-world situations where annotations are incomplete or unavailable [32].

Despite advances in supervised multimodal learning, there is a need to understand the behavior of these signals in unsupervised settings where no prior labels are available. Unsupervised analysis can provide information on the natural separability of individuals or activities based solely on the structure of the data, without model-driven bias. This is particularly relevant in scenarios involving unstructured data collection or personalized systems that must adapt to new users and contexts.

In this study, we performed a comparative evaluation of the ECG and ACC signals, individually and in combination, to recognize unsupervised activities of daily living and the identity of the subject. Using data collected from 30 participants during typical activities of daily living, such as walking, resting, working, climbing stairs, and lying down, we apply signal comparison and clustering methods to investigate (1) which modality is more effective in distinguishing between subjects or activities, and (2) whether multimodal fusion enhances or degrades clustering performance.

The paper is organized as follows. Section 2 reviews the existing literature on recognition of human activity and subject identification using ACC and ECG data. Section 3 describes the research methodology, including the data acquisition process, data comparison techniques, and clustering methods. The experimental results obtained by applying the methodology to data from 10 randomly selected individuals performing six activities are presented in Section 4. Section 5 discusses the findings, and finally, Section 6 concludes the paper and outlines directions for future research.

## 2. Related Work

In the literature, research efforts on recognition tasks involving physiological and motion signals can be broadly categorized into three main groups: studies focusing on ECG-based recognition, those focusing on ACC-based recognition, and approaches that combine ECG and ACC data. The following subsections review representative work in each category, highlighting key methodologies, datasets, and performance outcomes relevant to biometric identification and recognition of human activity.

However, these studies predominantly rely on supervised learning methods and annotated datasets. In contrast, our work introduces an unsupervised approach that does not require labeled data, aiming to explore the potential of multimodal physiological signals for activity and identity recognition in unlabeled real-world scenarios. The following subsections review representative works in each category to situate our contribution within the existing landscape.

### 2.1. ACC-Based Recognition

A study of four publicly available human activity datasets is presented in [18]. Data were collected from various IMU sensors, including a 6-axis motion tracking device equipped with a triaxial gyroscope and a triaxial accelerometer. The study investigates how combining accelerometer and gyroscope data at different fusion levels affects classification performance in HAR systems. The results clearly indicate that data fusion improves accuracy, and decision-level fusion achieves the best results across all four datasets.

A novel method for human activity recognition, based on the combination of high-gain observer and deep learning-based classification algorithms, is presented in [7]. The approach exploits the non-linear dynamic behavior of IMU sensors to design an observer for accurate attitude estimation. The estimated attitude is then used to generate spectrogram images representing different human activities. These images are used as input to train a deep learning-based computer vision model. Experimental results, based on over 750 min of labeled data, show that incorporating an observer-based attitude significantly improves the accuracy of the activity recognition system.

In [15], the authors integrate multiview fusion with contrastive loss to improve human activity classification using ACC data. The method combines both temporal and spectral views of the data through an attention mechanism that enhances the model’s ability to interpret activity patterns. To improve the separability of learned representations across activity classes, a siamese network based on contrastive loss is incorporated. When evaluated in the harAGE dataset [33], the proposed approach demonstrates a significant improvement over existing state-of-the-art methods.

The study in [20] explores the use of ACC data from smartphones to analyze individual unique gait patterns for user identification. The STFT is applied to the signal to produce 2D time–frequency image representations, which are then used to train a CNN-based classifier. To assess the effectiveness of the proposed model, a real-world benchmark dataset is used. Its performance is compared with several baseline methods, including logistic regression, nearest neighbors, random forest, decision tree, and multilayer perceptron. The results show that the deep CNN-based approach outperforms these baselines across multiple performance metrics and also exceeds the performance of other CNN architectures in all the measures evaluated.

Another notable study is presented in [19]. It investigates the use of smartphone sensor data for user authentication based on behavioral patterns. The proposed method uses DeepResNeXt, a variant of deep residual networks, to accurately and efficiently recognize smartphone users. The model is trained on publicly available smartphone datasets and is evaluated against several state-of-the-art approaches. Experimental results show that the proposed framework outperforms existing benchmark models, confirming its superior effectiveness in user recognition tasks.

In [21], a new biometric approach to person identification is introduced, based on behavioral patterns in smartphone use (such as variations in device positioning during carrying, talking, and other daily activities). A subset of signals from a large-scale dataset, comprising accelerometer, gyroscope, and magnetometer data, is labeled using a one vs. all classification strategy. Feature extraction is followed by classification using machine learning techniques, specifically k-Nearest Neighbors (kNN) and Randomized Neural Networks (RNN). The results confirm that behavior-based biometric identification using smartphone accelerometer data can achieve very high accuracy.

Most ACC-based studies similarly rely on labeled datasets and supervised classification models. Unlike these approaches, our work explores unsupervised clustering of accelerometer signals to assess their standalone and combined discriminative power.

### 2.2. ECG-Based Recognition

The study presented in [11] employs ECG signals as the only input modality for the dual tasks of classification of physical activity and biometric verification. The data analyzed originate from two publicly available ECG datasets (from clinical-grade and wearable devices). Adaptive techniques based on prior activity classification are proposed to improve biometric performance. The findings indicate that the framework can perform activity recognition and biometric verification in different device categories. Building on this dual-task approach, You et al. [13] introduce a more advanced deep learning architecture—DT4ECG—designed for human identification and activity recognition based on ECG signals. The model employs a robust one-dimensional convolutional neural network (CNN) architecture enhanced with residual blocks to extract informative and discriminative features from the data. Experimental evaluations demonstrate the effectiveness of the model, achieving high accuracy in both identification and activity recognition tasks.

Researchers in [12] explore the use of single-lead ECG signals recorded by a non-medical wearable ECG shirt to recognize human activity. Data are collected during four daily activities: sleeping, sitting, walking, and running. The signals obtained are transformed into spectrograms using the Short-Time Fourier Transform (STFT), which serve as input to a CNN. The results suggest that the ECG signals collected from wearable devices have informative value for HAR. The authors indicate plans to incorporate additional sensors into future work to improve the accuracy of recognition.

In [10] a comprehensive ECG-based biometric survey is presented. Provides an overview of the preprocessing, feature extraction, and transformation techniques of ECG signals used in biometric systems, together with a summary of publicly available ECG databases. In addition, it discusses a variety of classification methods, including deep supervised learning, semisupervised, and unsupervised learning. Finally, the authors outline modern strategies for multimodal integration of biometric data. Similarly, a promising new method for ECG-based human recognition is introduced in [6]. The method, named Collaborative-Set Measurement (CSM), performs distance measurement on multi-set bundles composed of multiple sample sets acquired under varying conditions. CSM demonstrates both effectiveness and efficiency by achieving high accuracy when evaluated in the DREAMER public database. Moreover, the method shows strong potential for use in scenarios with various data-degrading artifacts, such as those caused by motion, physiological conditions, or emotional states. Moreover, in [5], the authors propose an ECG-based human identification system that uses combined temporal-amplitude characteristic vectors. Fiducial points are used to generate feature vectors, and similarity is assessed through distance-based evaluation. The experimental results on data from 100 individuals show a high recognition rate. Furthermore, the findings confirm that recognition performance improves proportionally with the number of input heartbeats.

Although most studies demonstrate the effectiveness of ECG signals in supervised biometric and activity recognition settings, they do not explore unsupervised techniques or evaluate performance without labeled data. Our study addresses this gap by investigating ECG signals in a clustering-based framework.

### 2.3. ECG and ACC Combination-Based Recognition

Multimodal wearable sensors (ACC and ECG) are used in [24] to recognize human activities, including seven daily movements (from healthy subjects) and four restricted activities performed under medical supervision (by patients). Recognition is performed using various deep neural networks, including CNN, as well as unidirectional and bidirectional variants of long- and short-term memory (LSTM) and gated recurrent unit (GRU) networks. The results show that combining convolutional feature extraction and bidirectional sequential modeling enables effective learning of features from multimodal time-series data.

CNNs are applied for the purpose of human activity recognition in [23]. Data from ten healthy subjects are recorded using a five-lead wireless ECG, a 3D accelerometer, and a 3D gyroscope. The proposed approach achieves high accuracy in a wide range of activities, including jumping, running, sitting, climbing stairs, walking, bending over, and standing up. The improved recognition accuracy is attributed to the combination of inertial sensors and ECG leads.

In [25], a patch-type flexible sensor is used that integrates both ECG and ACC signals. Data were recorded from 20 healthy volunteers and 25 patients performing seven different activities. Human activity recognition was conducted using several deep learning models, including CNN, LSTM, CNN-LSTM, and GRU. The results demonstrate that the combination of ECG and ACC data significantly improves the accuracy of HAR compared to the use of ACC data alone.

The identification of individuals based on data recorded from a wireless ECG and motion sensors is presented in [27]. In the experimental setting, 34 participants were examined during seven different physical activities. The signals were captured synchronously by a 5-lead wireless ECG, two 3D accelerometers, and a 3D gyroscope. The features of the time and frequency domain were used to train a multilayer CNN, which achieved high classification accuracy. In addition, activity recognition was performed to demonstrate the varying physical demands of the analyzed activities. The resulting accuracy was also high.

In [26], biometric verification was carried out using a prototype composed of low-cost wearable sensors, including photoplethysmogram (PPG), ECG, ACC, and galvanic skin response (GSR) sensors. Data were collected from 25 participants in three conditions: sitting at rest, walking, and sitting after light physical activity. Various combinations of the recorded signals were analyzed to assess their effectiveness in biometric verification.

The patent [28] describes a system and method for identifying users through a wearable device that captures both motion data (from accelerometers and gyroscopes) and heartbeat waveform data. By integrating these two types of biometric input, the system improves the precision and reliability of user authentication. Machine learning models, such as support vector machines (SVMs) and neural networks, are used to analyze the combined data and verify the user’s identity over time.

Most existing studies focus on supervised classification models that require labeled data to guide learning. Unlike these works, our study adopts an unsupervised approach to assess the ability to cluster daily activities and identify individuals based on patterns in ACC and ECG signals, both separately and combined. Although prior work emphasizes optimizing classification accuracy with deep learning or feature engineering in specific datasets, we analyze real-world data without labels to determine whether multimodal fusion enhances or impairs unsupervised clustering performance. This approach highlights the complementary strengths and limitations of each modality in practical unlabeled scenarios, offering new insights for developing more adaptable and robust recognition systems.

## 3. Materials and Methods

**Figure 1:**
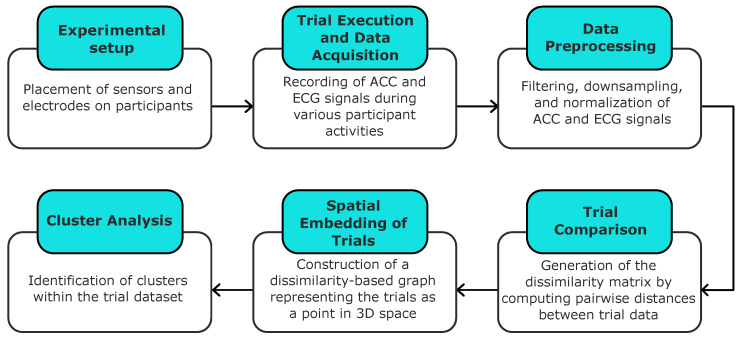
A schematic overview of the proposed methodology.

In this section, we present the methodology adopted in this study, which includes signal acquisition and preprocessing, pairwise comparison of recorded trials, spatial embedding of the data, and subsequent cluster analysis. An overview of the main steps involved in the proposed framework is presented in Figure 1.

### 3.1. Data Acquisition Protocol

This study reuses the dataset originally collected by Sidek et al. [9], which was designed to evaluate human activity in a real-world setting. The experiments were conducted in a single-family house with stairs, where 30 volunteers (19 women, 11 men; age range: 18–81 years) performed six selected activities of daily living. The demographic characteristics of the participants are summarized in Table 1.

**Figure 2:**
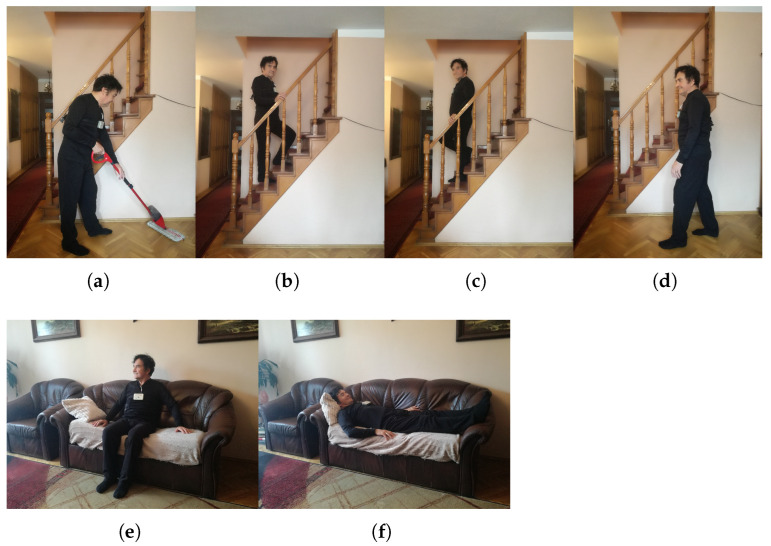
Performed activities of daily living: (**a**) Working. (**b**) Going upstairs. (**c**) Going downstairs. (**d**) Walking. (**e**) Lying on the couch. (**f**) Resting.

The activities performed include (1) working (cleaning the floor), (2) going upstairs, (3) going downstairs, (4) walking, (5) lying on the couch, (6) resting (sitting on the couch with a very low intensity of body movements). Figure 2 presents the chosen video frames that illustrate the activities mentioned above.

Physiological signals were acquired using the Revitus ECG and ACC module, a compact wireless wearable device capable of simultaneously recording ECG signals and 3D ACC data. The module is powered by a 1300 mAh rechargeable battery and is attached to the participant’s chest using an adjustable elastic strap, ensuring secure placement during movement. The A/C processing has a 12-bit resolution over a input range of ±10 mV.

**Figure 3:**
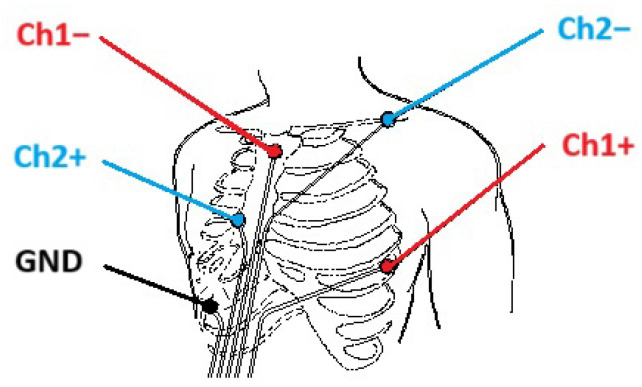
Placement of ECG electrodes for two bipolar leads; adapted from [8,9,34,35].

ECG signals were recorded from two bipolar leads at a sampling rate of 1000 Hz, while triaxial ACC data were collected at 100 Hz. The electrodes were placed according to a predefined configuration, as illustrated in Figure 3. The first lead was placed with the positive electrode in the fifth intercostal space along the anterior axillary line and the negative electrode on the right side of the manubrium. The second lead had its positive electrode aligned with the fourth intercostal space in the sternum and the negative electrode in the left subclavian region. In addition, a ground electrode was placed in the fifth intercostal space on the midaxillary line.

**Figure 4:**
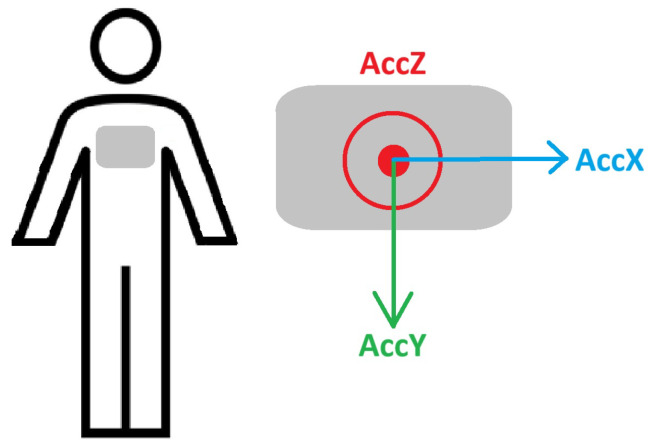
Reference coordinate system used for ACC signal acquisition.

The ACC module is built inside the Revitus device; therefore, it measures the ACC signals from the center of the participant’s chest. The direction and orientation of each of the ACC coordinates are presented (from a frontal view) in Figure 4.

During signal acquisition, data were transmitted to a nearby notebook computer via Bluetooth. Transmission could be performed in real time or delayed after local storage in the device’s internal memory, depending on the selected operational mode. In our studies, the real-time transmission mode was employed to enable continuous monitoring of the signals during recording.

### 3.2. Signal Preprocessing

After the experimental sessions were completed, the recorded signals were transferred to a computer and analyzed using MATLAB R2024b (MathWorks, Natick, MA, USA). ACC and ECG signals were subjected to pre-processing steps to reduce noise and prepare the data for subsequent analysis and comparison.

ACC signals were processed using a fourth-order low-pass Butterworth filter with a cutoff frequency of 6 Hz to preserve low-frequency components related to movement while attenuating high-frequency noise and irrelevant fluctuations.

ECG signals, originally sampled at 1000 Hz, were first filtered using a fourth-order zero-phase Butterworth bandpass filter with cutoff frequencies of 5–15 Hz to remove baseline drift and high-frequency noise, preserving the frequency range relevant to cardiac activity. Subsequently, the signals were downsampled to 500 Hz through decimation, incorporating appropriate low-pass filtering to mitigate aliasing and ensure signal fidelity. Finally, ECG signals underwent min-max normalization to reduce amplitude variability caused by differences in electrode placement, skin conductivity, and other physiological or environmental factors.

Finally, the quality of the ACC signals was assessed by visual inspection. Based on this evaluation, a representative 7 s segment of the preprocessed ACC and ECG signals was selected for further analysis.

### 3.3. Pairwise Trial Comparison

A dataset was constructed from the recorded and preprocessed data by randomly selecting 10 subjects, including a single repetition of each activity per subject. This procedure resulted in a total of 60 trials, with each subject contributing one instance of all six activity types. Each trial comprises five pre-processed time-series signals: three ACC signals along the orthogonal axes and two ECG signals acquired from separate channels. Although all signals were temporally aligned to cover the same recording duration, differences in their original sampling rates led to time series of unequal lengths. As a result, each trial is represented as a structured set of five temporally synchronized signals with varying temporal resolutions.

(1) $$S=\left[\begin{array}{ccccc} {\mathrm{ACC}}_{x}^{\left(1\right)} & {\mathrm{ACC}}_{y}^{\left(1\right)} & {\mathrm{ACC}}_{z}^{\left(1\right)} & {\mathrm{ECG}}_{1}^{\left(1\right)} & {\mathrm{ECG}}_{2}^{\left(1\right)}\\ {\mathrm{ACC}}_{x}^{\left(2\right)} & {\mathrm{ACC}}_{y}^{\left(2\right)} & {\mathrm{ACC}}_{z}^{\left(2\right)} & {\mathrm{ECG}}_{1}^{\left(2\right)} & {\mathrm{ECG}}_{2}^{\left(2\right)}\\ \vdots & \vdots & \vdots & \vdots \\ {\mathrm{ACC}}_{x}^{\left(i\right)} & {\mathrm{ACC}}_{y}^{\left(i\right)} & {\mathrm{ACC}}_{z}^{\left(i\right)} & {\mathrm{ECG}}_{1}^{\left(i\right)} & {\mathrm{ECG}}_{2}^{\left(i\right)}\\ \vdots & \vdots & \vdots & \vdots & \vdots \\ {\mathrm{ACC}}_{x}^{\left(N\right)} & {\mathrm{ACC}}_{y}^{\left(N\right)} & {\mathrm{ACC}}_{z}^{\left(N\right)} & {\mathrm{ECG}}_{1}^{\left(N\right)} & {\mathrm{ECG}}_{2}^{\left(N\right)} \end{array}\right]$$

where each element *i* of S corresponds to one trial and *N* is the total number of trials.

To assess the degree of similarity between ACC and ECG signal patterns across different activities and subjects, a pairwise comparison was performed on the entire dataset, resulting in 1770 unique trial combinations. The comparison pipeline consists of three main stages, which are detailed in the following.

#### 3.3.1. Cross-Correlation-Based Temporal Alignment

For each pair of trials indexed by i,j∈{1,…,N}, the normalized cross-correlation function between homologous signals (e.g., ${\mathrm{ACC}}_{x}^{\left(i\right)}$ and ${\mathrm{ACC}}_{x}^{\left(j\right)}$) was calculated to determine the lag $\tau^{*}$ that maximizes their linear similarity:

(2) $$\tau^{*}=\arg\max_{\tau }\sum_{t}x^{\left(i\right)}\left(t\right)\cdot x^{\left(j\right)}\left(t+\tau \right)$$

where $x^{\left(i\right)}$ and $x^{\left(j\right)}$ represent instances of the same signal type from trials *i* and *j*, respectively; *t* denotes the discrete time index, and τ is the lag parameter.

Subsequently, the signal $x^{\left(j\right)}$ was temporally shifted by the optimal lag $\tau^{*}$ to maximize alignment with $x^{\left(i\right)}$. Following alignment, both signals were truncated at their limits by discarding up to 5% of the total length, selectively removing samples exhibiting the highest pointwise dissimilarity. This trimming step mitigates the impact of poorly aligned or noisy segments, thereby enhancing the robustness of the subsequent similarity analysis.

#### 3.3.2. Dynamic Time-Warping Distance Computation

After applying temporal alignment and adaptive trimming, the pairwise similarity between trials was evaluated using the dynamic time-warping (DTW) algorithm. For each pair i,j∈{1,…,N}, the DTW was calculated between the corresponding signal instances $x^{\left(i\right)}$ and $x^{\left(j\right)}$ of the same signal type, using the Euclidean distance as the local cost function in the DTW algorithm. As each trial contains five signal types, this procedure results in a vector of five DTW distances for each trial pair:

(3) $$d^{\left(i,j\right)}=\left[\begin{array}{c} d\left(x_{{\mathrm{ACC}}_{x}}^{\left(i\right)},\;x_{{\mathrm{ACC}}_{x}}^{\left(j\right)}\right)\\ d\left(x_{{\mathrm{ACC}}_{y}}^{\left(i\right)},\;x_{{\mathrm{ACC}}_{y}}^{\left(j\right)}\right)\\ d\left(x_{{\mathrm{ACC}}_{z}}^{\left(i\right)},\;x_{{\mathrm{ACC}}_{z}}^{\left(j\right)}\right)\\ d\left(x_{{\mathrm{ECG}}_{1}}^{\left(i\right)},\;x_{{\mathrm{ECG}}_{1}}^{\left(j\right)}\right)\\ d\left(x_{{\mathrm{ECG}}_{2}}^{\left(i\right)},\;x_{{\mathrm{ECG}}_{2}}^{\left(j\right)}\right) \end{array}\right]$$

where $d^{\left(i,j\right)}$ denotes the DTW distance vector computed between the trials *i* and *j*.

#### 3.3.3. Distance Normalization and Aggregation

To allow meaningful aggregation of the calculated distances for each pair of trials, each component of the DTW distance vector $d^{\left(i,j\right)}$ was normalized to the interval [0,1], using the global minimum and maximum values observed across all pairs of trials, independently for each signal type, as defined by:

(4) $$d_{\mathrm{norm},k}^{\left(i,j\right)}=\frac{d_{k}^{\left(i,j\right)}-d_{k,\min}}{d_{k,\max}-d_{k,\min}}\;\mathrm{for}\;k=1,\ldots ,5$$

where $d_{k}^{\left(i,j\right)}$ denotes the DTW distance for the signal *k*-th between the trials *i* and *j*, and $d_{k,\min}$, $d_{k,\max}$ are the minimum and maximum values, respectively, observed in all comparisons for that signal type.

The general dissimilarity between two trials, denoted as $D^{\left(i,j\right)}$, was then calculated as the Euclidean norm of the normalized DTW distance vector.

(5) $$D^{\left(i,j\right)}=∥d_{\mathrm{norm}}^{\left(i,j\right)}∥=\sqrt{\sum_{k=1}^{5}{\left(d_{\mathrm{norm},k}^{\left(i,j\right)}\right)}^{2}}$$

This approach assigns equal importance to all signal types, ensuring a balanced contribution from each modality in the final dissimilarity measure. The procedure was applied to all unique trial pairs, producing a dissimilarity matrix that captures the pairwise similarity structure throughout the dataset.

### 3.4. Spatial Embedding of Trials

Following the construction of the dissimilarity matrix, trials are embedded in a 3D space by generating a weighted undirected graph. In this graph, each node corresponds to a distinct trial, while edge weights encode pairwise dissimilarities between trials. This transformation effectively converts temporally dependent data into a spatial arrangement suitable for visualization and subsequent multivariate analysis.

To determine the layout of the graph, a force-directed drawing algorithm is employed, which iteratively adjusts the positions of the nodes by simulating forces of attraction and repulsion based on the dissimilarity values. The goal of this process is to minimize the overall energy of the system, resulting in an intuitive spatial arrangement of the trials [36,37].

In this fully connected graph, every pair of nodes (trials) influences the final layout through these forces, defined as:

(6) $$\begin{array}{cc} f_{r}\left(i,j\right) & =\frac{-CK^{2}}{∥y(i)-y(j)∥}\;e^{\alpha |D(i,j)|} \end{array}$$

(7) $$\begin{array}{cc} f_{a}\left(i,j\right) & =\frac{{∥y\left(i\right)-y\left(j\right)∥}^{2}}{K}\;e^{\frac{\gamma }{\left|D(i,j)\right|}} \end{array}$$

where $f_{r}\left(i,j\right)$ and $f_{a}\left(i,j\right)$ denote the repulsive and attractive forces between the nodes *i* and *j*, respectively; y(i) and y(j) represent their coordinates in the embedding space; and D(i,j) is the measure of dissimilarity between the corresponding trials.

In Equations (6) and (7), the parameter *K* defines the spring length that governs the equilibrium distance between the nodes, while *C* controls the relative magnitude of the repulsive force. The constants α and γ are scaling factors that regulate the exponential sensitivity of the repulsive and attractive forces, respectively. The resulting combined force between a pair of nodes is given by:

(8) $$f_{c}\left(i,j\right)=f_{r}\left(i,j\right)+f_{a}\left(i,j\right)$$

The final layout of the graph is obtained through multiple iterations, during which nodes representing similar trials (i.e., with low dissimilarity) are drawn together by attractive forces, while dissimilar trials are pushed apart by stronger repulsive forces. This dynamic process drives the system toward a low-energy configuration that reflects the structure encoded in the dissimilarity matrix.

As a result, the graph self-organizes into a stable 3D configuration, where Euclidean distances serve as effective approximations of the original dissimilarity values. This emerging spatial topology allows for the identification of similarity patterns by revealing clusters between trials, providing a solid foundation for subsequent patterns analysis, classification, and clustering tasks [38].

### 3.5. Cluster Analysis

To identify natural clusters among the embedded trials, Hierarchical Agglomerative Clustering (HAC) was applied to the 3D spatial layout derived from the dissimilarity matrix. This unsupervised method constructs a hierarchy by iteratively merging the most similar pairs of elements based on a specified distance metric [39]. Although HAC does not inherently require a predefined number of clusters, in this study the desired number was explicitly specified to align with the expected categories (e.g., subjects or activities), allowing for direct comparison with ground-truth labels and subsequent evaluation using clustering performance metrics.

Instead of clustering in a single step, a staged approach was implemented to refine the separation of the clusters. At each stage, the data were initially divided into two candidate clusters. If the silhouette coefficient (SC) exceeded 0.5, the smaller cluster was preserved as a final cluster, while the larger cluster was recursively re-analyzed in the next stage. This new stage involved re-computing pairwise trial dissimilarities, embedding the trials in a 3D space, and applying HAC again. The process continued until the number of identified clusters matched the target (e.g., six clusters for activity recognition or ten clusters for subject identification). When SC fell below 0.5, suggesting insufficient separation between two clusters, the procedure was terminated by imposing a direct partition into the remaining number of clusters required to reach the target.

**Figure 5:**
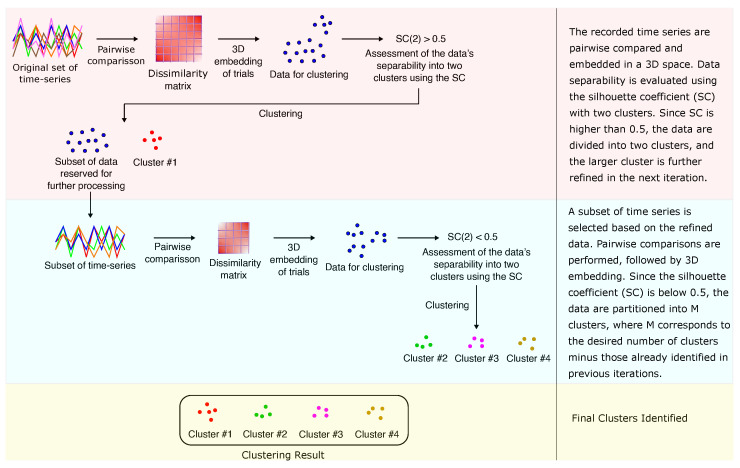
Illustration of the staged clustering refinement procedure.

This staged refinement is illustrated in Figure 5. In the example, two clusters are obtained in the first stage since the SC evaluated at k=2 was greater than 0.5. The smaller cluster is retained, while the larger one proceeds to a second stage. There, the SC was below 0.5, and therefore the remaining number of clusters (three in this case, assuming a total of four was desired) was directly assigned. This ensures that the final number of clusters is equal to the expected number of ground-truth groups, which is essential for the subsequent computation of classification-oriented metrics after optimal label alignment using the Hungarian algorithm [40].

Cluster performance was evaluated using a set of standard metrics: SC to assess intra- and inter-cluster compactness; the normalized mutual information (NMI) to measure agreement with known labels (subjects or activities); and accuracy, recall, and F1 score to evaluate classification equivalence.

## 4. Results

**Figure 6:**
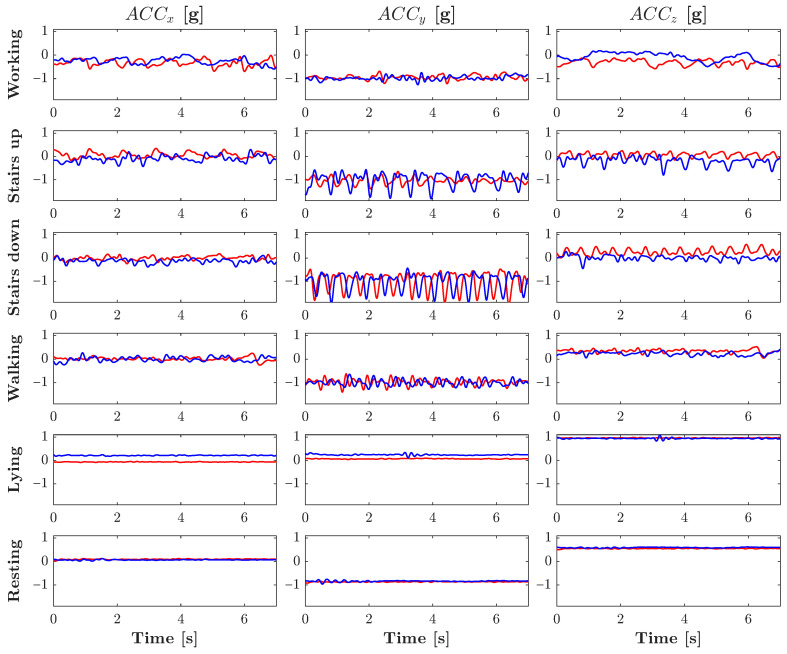
Preprocessed ACC signals recorded from two representative participants during the performed activities.

**Figure 7:**
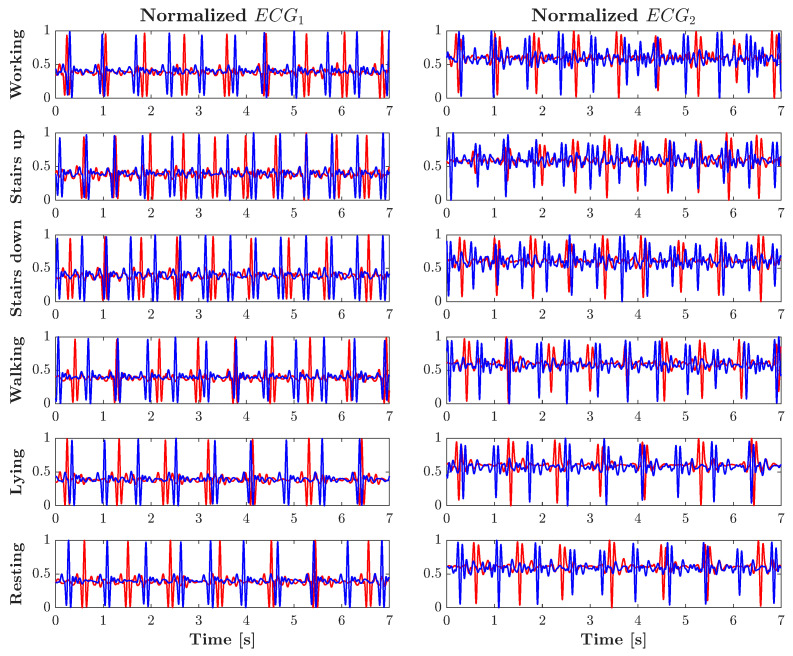
Preprocessed ECG signals recorded from two representative participants during the performed activities.

The results obtained using the proposed methodology are presented and discussed in this section. Figure 6 shows the preprocessed ACC signal waveforms recorded from two representative participants during the activities carried out. Consequently, Figure 7 shows the corresponding pre-processed ECG signals for the same participants. Notable differences are observed in the patterns of amplitude and frequency between subjects and activities, particularly in the ACC signals, highlighting the unique physiological and behavioral characteristics captured by each modality.

Under static conditions, the gravitational acceleration detected by the accelerometer provides a reliable reference for assessing the body’s spatial orientation and posture. Visual inspection of ACC signals (see Figure 6) reveals that lying and resting produce waveforms with low variability and minimal oscillatory components, due to the limited movement involved. In contrast, dynamic activities exhibit distinct dominant frequencies that vary not only between activity types but also between participants, suggesting greater complexity in discriminating these patterns solely through visual analysis.

Among all recorded tasks, the negotiation of stairs produces the highest overall acceleration amplitudes, reflecting the increased physical effort and vertical displacement involved.

In both walking and stair ascent or descent, the acceleration measured along the y-axis is dominant, capturing the vertical motion of lifting or lowering the body against gravity. In contrast, the acceleration components along the x-axis (mediolateral) and z-axis (anteroposterior) remain relatively low, indicating limited lateral and forward motion.

In contrast, working generates the strongest accelerations along the x- and z-axes, corresponding to side-to-side torso rotation and forward-reaching motions. The y-axis shows only minor variation, indicating limited vertical movement during this task.

Visual inspection of ECG signals (see Figure 7) reveals that although the overall waveform pattern remains consistent between different activities when recorded from the same channel, there is noticeably less variability between signals from the same subject compared to signals from different subjects.

To complement the arguments extracted from the qualitative inspection of the recorded signals, a set of quantitative features was extracted from the recordings. For each of the 33 participants, the mean, standard deviation, and dominant frequency of the signals were calculated for the six activities considered. These values were then averaged among participants to characterize the patterns at the group level.

**Figure 8:**
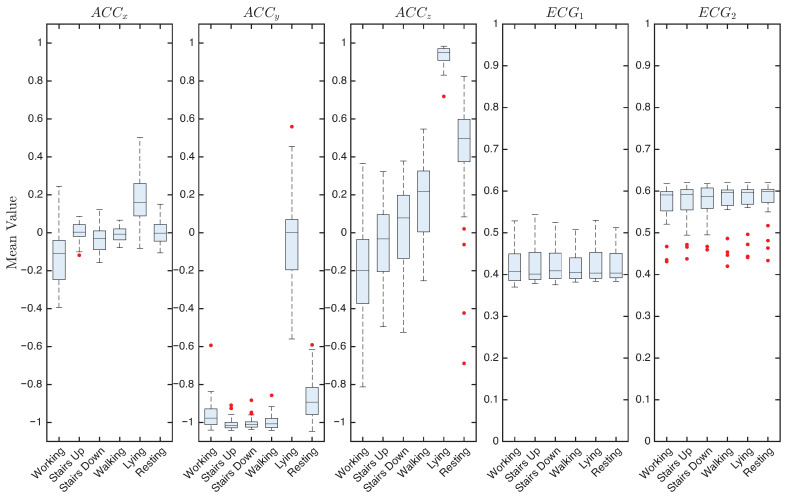
Mean value of ACC and ECG signals across the six activities, averaged over 33 participants. Red dots represent outliers.

Figure 8 shows the distribution of the mean signal values, with accelerations expressed in units of *g* and ECG signals normalized in the range [0, 1]. Accelerations along the x-axis remain close to zero in all activities, showing minimal variability between subjects. The y-axis also shows low variability, with accelerations centered on the expected reference value of −g for most activities. The interquartile range (IQR), which represents variability between participants, is generally narrow, although a few outliers are observed, likely attributable to differences in sensor placement or body orientation. A notable exception occurs in lying activity, where the variability between participants increases and the mean value approaches zero. In contrast, the z-axis exhibits the greatest variability between subjects and activities, with a substantial number of outliers observed particularly during resting.

Regarding ECG signals, channel 1 shows a consistently low IQR with negligible outliers, while channel 2 shows a larger number of outliers. However, inter-subject variability (captured by the IQR) and interactivity variability (reflected in shifts of median values) remain limited. The mean values are approximately 0.40 for channel 1 and 0.58 for channel 2 across all activities, indicating a stable central tendency despite the presence of occasional outliers.

**Figure 9:**
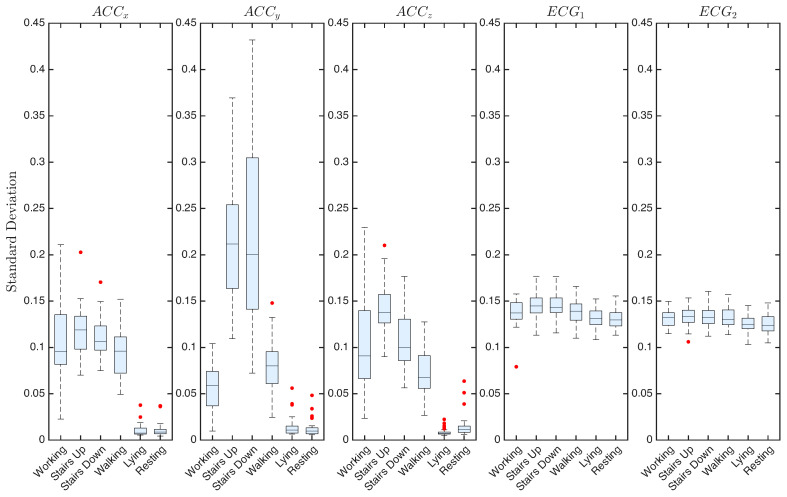
Standard deviation of ACC and ECG signals across the six activities, averaged over 33 participants. Red dots represent outliers.

In Figure 9, the standard deviation of the signals averaged across participants is shown. This representation enables the assessment of variability both between participants within each boxplot and across different activities when comparing them. For accelerations, static activities exhibit very low standard deviations, consistent with negligible movement, and the observed outliers remain close to the median. In contrast, dynamic tasks present greater variability, with $ACC_{y}$ reaching maximum median values of approximately 0.2 g during the ascent and descent of stairs. ECG signals, by comparison, maintain low variability in all tasks and participants, indicating that the changes remain subtle.

**Figure 10:**
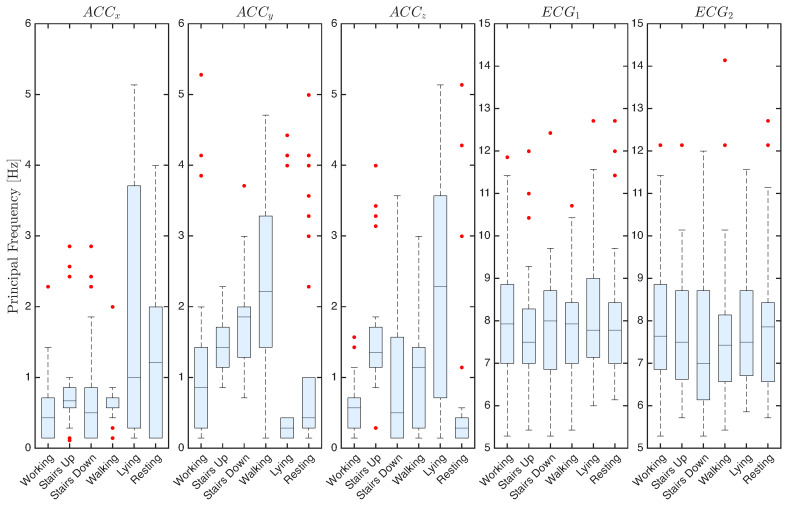
Dominant frequency of ACC and ECG signals across the six activities, averaged over 33 participants. Red dots represent outliers.

Figure 10 presents the dominant frequencies of the signals. For ACC signals, different principal frequencies are observed, mostly concentrated in the low-frequency range, although numerous outliers appear across all axes. Walking and stair-related activities reveal distinct frequency bands that align with the cadence during these activities. For ECG signals, the dominant frequencies in both channels remain around 7–8 Hz, with comparable median values across activities. Some of the observed dispersion, particularly in dynamic tasks, can likely be attributed to residual noise and motion artifacts.

### 4.1. Signal Modality Scenarios

The methodology applied in this study follows an unsupervised approach aimed at uncovering intrinsic patterns within the data by grouping similar trials without using predefined labels or ground-truth information. This means that the method does not explicitly perform classification tasks, such as identifying activities or subjects; instead, it clusters trials based solely on their inherent similarities. This flexibility allows for the exploration of different recognition possibilities. To this end, the analysis was performed using three different signal-modality scenarios, each based on distinct subsets of the recorded data.

#### 4.1.1. ACC-Based Recognition

The results obtained from the implementation of the proposed methodology, considering only the ACC signals, are presented below.

**Figure 11:**
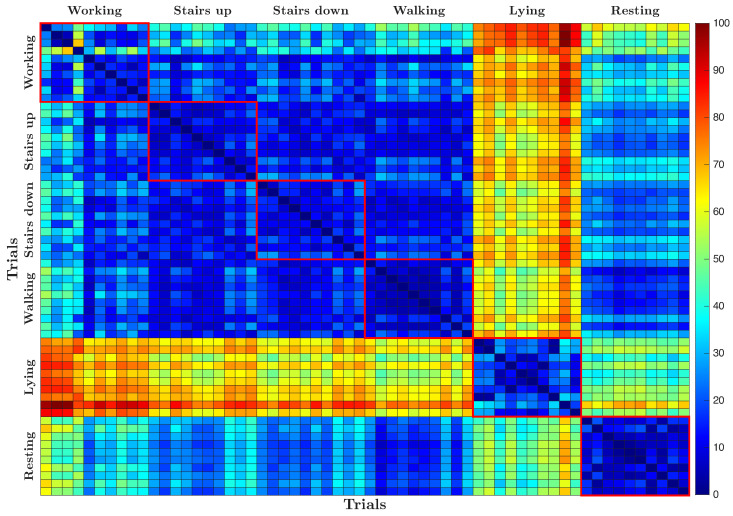
Dissimilarity matrix computed from pairwise comparison between ACC signals recorded during the different trials.

Figure 11 shows the dissimilarity matrix resulting from the pairwise comparison of all recorded trials, where each cell represents the calculated dissimilarity measure between two trials. The matrix is visualized as a grid of colored cells along with a color scale that maps numerical values to a gradient ranging from blue to red. Lower dissimilarity values between trials are shown in cooler blue tones, while higher values are shown in warmer red tones. As expected, the diagonal reflects the comparison of each trial with itself, which naturally results in the minimum dissimilarity value of zero, and appears as the darkest blue.

The dissimilarity matrix reveals that lying activity is the most different from all other activities, as evidenced by the warmer colors indicating higher pairwise dissimilarity values. Resting also shows a degree of differentiation, although less pronounced compared to lying. In contrast, activities such as working, climbing stairs, scaling stairs, and walking generally exhibit lower dissimilarity values among themselves, represented by cooler colors in the matrix. In particular, working shows a slight tendency to diverge from the other dynamic activities, indicating subtle differences in the underlying signal patterns.

**Figure 12:**
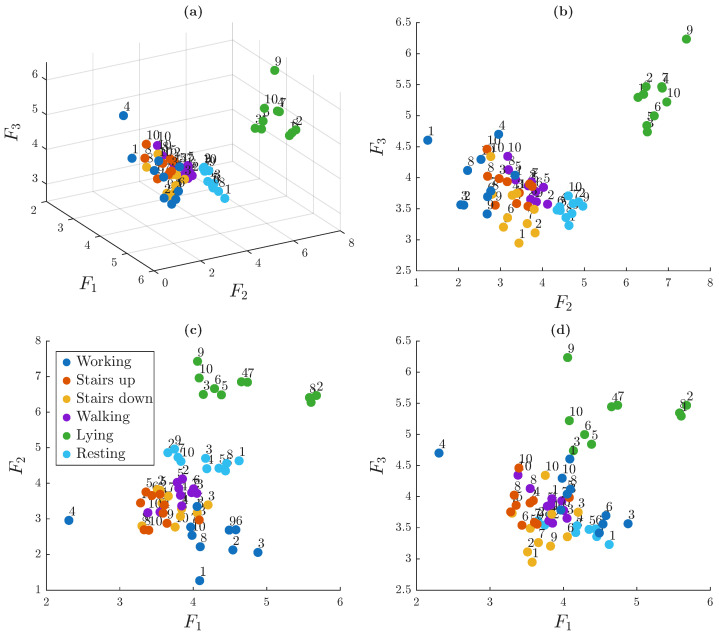
Three-dimensional spatial embedding of trials based on the dissimilarity matrix. Views shown: (**a**) isometric (view angled at 60° azimuth and 30° elevation), (**b**) frontal, (**c**) top, and (**d**) lateral.

Figure 12 presents the 3D embedding of the 60 trials, derived from the dissimilarity matrix, which visually reinforces the structure observed previously. Multiple views are displayed to better illustrate the spatial relationships. Each node in the graph represents a single trial, identified by a numerical label corresponding to the individual that performed the trial and colored according to the associated activity.

Distinct clusters are visible, most notably for lying activity (light green nodes) and, to a lesser extent, for resting activity (light blue nodes). The trials corresponding to the working activity also form a relatively compact group. In contrast, trials related to walking, ascending stairs, and descending stairs are positioned closer to each other, suggesting a higher degree of similarity among these dynamic activities. Importantly, there is no apparent grouping based on individual subjects, indicating that ACC-based signals do not retain strong person-specific signatures under unsupervised analysis.

To automate the identification of clusters in the generated 3D embedding space, a hierarchical clustering strategy was used in five consecutive stages. At each stage, the current data subset was split into two clusters and only the larger cluster was selected for further subdivision in the next stage. In the final stage, this remaining subset was divided into three clusters. As a result, a total of six distinct clusters were obtained through this progressive data-driven refinement.

**Figure 13:**
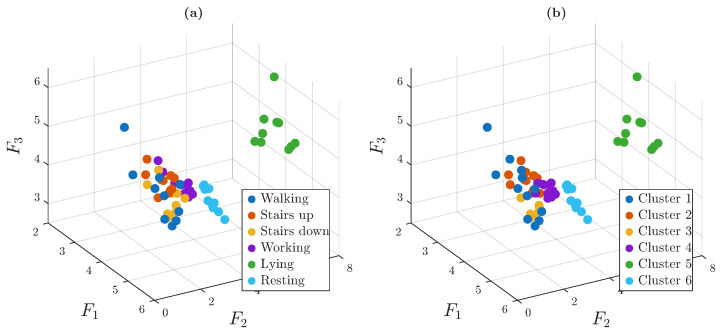
Clustering results obtained through a five-stage clustering process. (**a**) Graph view with ground-truth activity labels. (**b**) Graph view with cluster assignments.

Figure 13 presents the results of the application of HAC. The plot on the left shows an isometric view of the 3D embedded space, where each node is colored according to the ground-truth activity labels. The right plot shows the same embedding, but with nodes colored according to the cluster assignments.

The resulting cluster structure aligns well with the actual activity categories, indicating that the proposed approach effectively captures meaningful patterns in the data.

#### 4.1.2. ECG-Based Recognition

The results obtained from the implementation of the proposed methodology, considering only the ECG signals, are presented below.

**Figure 14:**
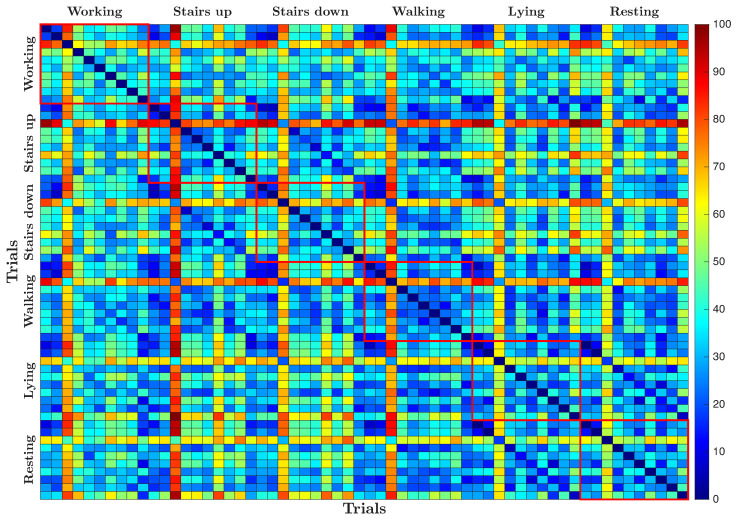
Dissimilarity matrix computed from pairwise comparison between ECG signals recorded during the different trials.

Figure 14 shows the dissimilarity matrix computed from the ECG signals. Unlike the well-defined block regions seen in the ACC-based dissimilarity matrix, this matrix presents distinct horizontal and vertical warm-colored lines. These patterns indicate that the main differences in the ECG data come from variations between subjects rather than from the activities performed.

The repeated lines across the matrix reflect consistent and subject-specific cardiac signatures, while the lack of clear separation by activity suggests that ECG signals have a limited ability to differentiate between different physical activities.

**Figure 15:**
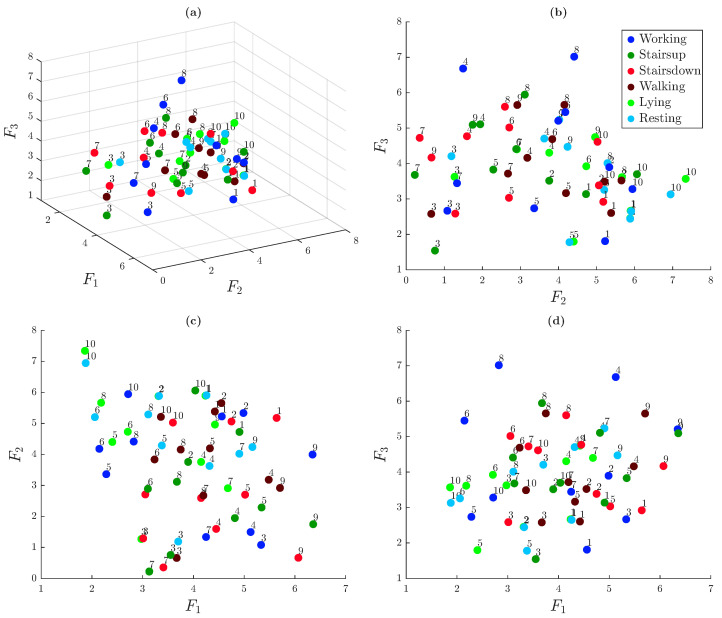
Three-dimensional spatial embedding of trials based on the dissimilarity matrix. Views shown: (**a**) isometric (view angled at 60° azimuth and 30° elevation), (**b**) frontal, (**c**) top, and (**d**) lateral.

Figure 15 presents the spatial embedding of the trials based on pairwise comparisons of ECG signals derived from the dissimilarity matrix. Each node represents a single trial, labeled by the participant number and colored according to the associated activity.

The cluster formed by the trials of participant 3 is clearly visible, as their ECG signals differ noticeably from those of the other participants. These trials are grouped close together in the same area and are separated from the others. In general, trials from the same person (nodes with the same label) tend to be near each other, even if they are spread out in different parts of the embedding space. This supports the idea that ECG signals are more useful for identifying individuals than for recognizing activities. However, it is also important to note that the clusters are not clearly separated in general, as many remain close to each other, suggesting a limited ability to distinguish some subjects using an unsupervised learning approach.

HAC was once again used to automatically categorize the clusters observed in the 3D embedding space. The clustering process was carried out in three stages. In the first and second stages, clearly separable clusters were isolated. In the final stage, the algorithm was set to identify eight clusters to match the number of subjects who conducted the trials.

**Figure 16:**
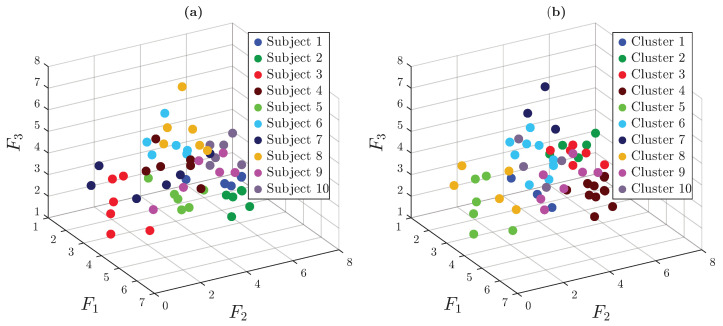
Final clustering outcome obtained through a three-stage clustering process. (**a**) Graph view with ground-truth activity labels. (**b**) Graph view with final cluster assignments.

Figure 16 shows that while several trials were misclassified, many were correctly grouped by subject. Misclassified trials are often close to each other in the embedding space, suggesting overlap or similarity in ECG patterns among some subjects.

These findings highlight the suitability of ECG signals for identifying individuals, while also highlighting the challenges inherent in differentiating physical activities based solely on cardiac dynamics.

#### 4.1.3. Combined ECG and ACC-Based Recognition

In this scenario, both the ECG and the ACC signals were jointly analyzed to investigate whether their combination improves the discrimination of physical activities and/or individual identities.

**Figure 17:**
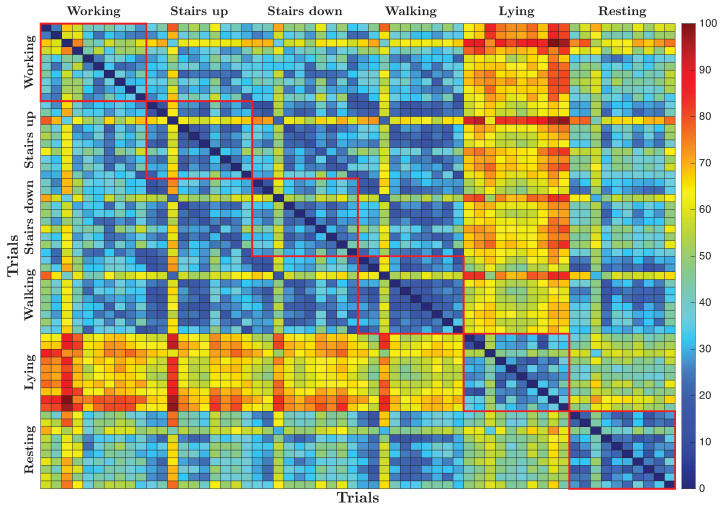
Dissimilarity matrix for combined ECG and ACC signals.

The dissimilarity matrix presented in Figure 17 exhibits patterns that are less clearly separated compared to the individual modalities. The matrix suggests that the resulting clusters do not correspond exclusively to either activities or subjects, but rather to a mixture of both factors.

**Figure 18:**
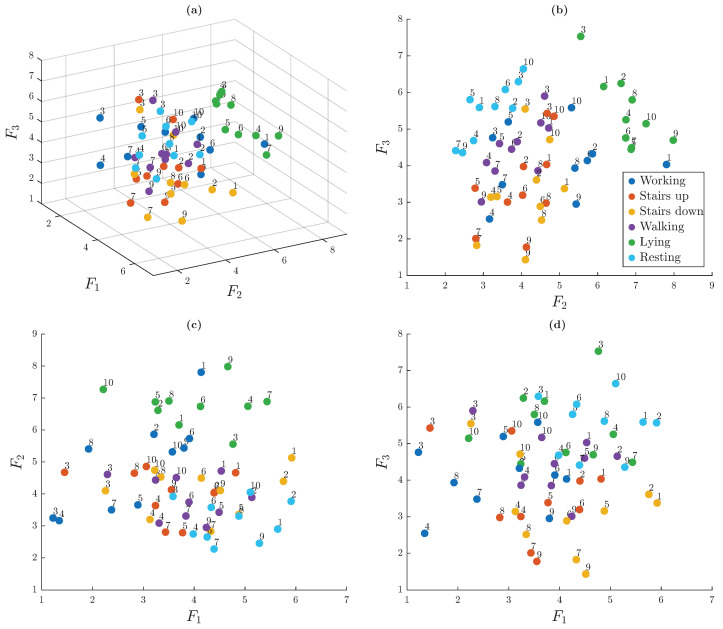
Three-dimensional spatial embedding of trials based on combined ECG and ACC dissimilarities. Views shown: (**a**) isometric (view angled at 60° azimuth and 30° elevation), (**b**) frontal, (**c**) top, and (**d**) lateral.

Figure 18 shows the spatial embedding of the trials in a 3D space. Unlike previous scenarios, some nodes form activity-based clusters, such as those associated with lying, while others appear to group trials from specific subjects, such as subject 3. In general, the clusters are more intermixed than in previous scenarios, suggesting that combining the modalities introduces overlapping patterns that make separation more difficult.

**Figure 19:**
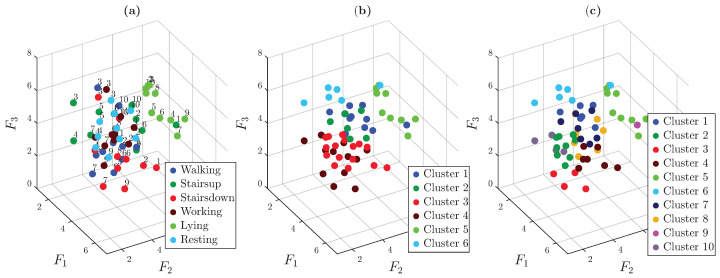
Clustering results obtained through a three-stage clustering process. (**a**) Graph view with ground-truth activity labels. (**b**) Graph view with six cluster assignments. (**c**) Graph view with ten cluster assignments.

Similarly to previous scenarios, HAC was used to explore whether the clusters visible in the 3D space could be automatically separated. Since it was unclear whether the combined ACC + ECG modality using the proposed methodology better groups activities or individuals, clustering was performed in three stages. In the first and second stages, the most identifiable clusters were separated from the rest, and in the final stage, either four or eight clusters were identified depending on whether the separation corresponded to activities or subjects, as shown in Figure 19.

These observations suggest that while the integration of ECG and ACC signals offers richer information, the increased dimensionality and potential confounding between subject and activity-related patterns may complicate clustering. Consequently, for certain recognition tasks, it may be advantageous to use the ECG and ACC modalities separately rather than in combination.

## 5. Discussion

This section discusses the implications of applying the proposed unsupervised methodology for recognizing physical activities or individuals using ACC and ECG data, separately and combined. The objective was to assess the contribution of each modality to revealing intrinsic data structures and to discuss the challenges arising from the integration of electrophysiological and motion-related data.

To derive meaningful conclusions from the analysis, the clustering performance was quantitatively evaluated using NMI and SC. NMI measures the amount of shared information between the clustering assignments and the ground-truth labels, indicating how well the clusters correspond to known categories. SC assesses the compactness and separation of clusters. Additionally, to enable a fair comparison with the true labels, an optimal mapping between predicted cluster labels and ground-truth classes was established using the Hungarian algorithm. This mapping allowed for the calculation of supervised classification metrics such as accuracy, F1 score, recall, and precision, providing further insight into cluster quality beyond unsupervised measures.

Given that ACC signals better differentiate between activities, performance metrics for this modality were calculated using ground-truth activity labels. In contrast, ECG signals are more suitable for distinguishing individual subjects, so their evaluation was based on the identities of the subjects. For the combined ACC + ECG modality, metrics were computed separately for both activities and subjects to assess performance in both contexts. Table 2 summarizes the performance metrics computed for each signal modality.

The analysis of ACC signals demonstrated clear potential for unsupervised activity recognition. The clustering method effectively separated static behaviors, such as lying and resting, from dynamic movements, such as walking and climbing stairs, producing well-defined clusters that align closely with ground-truth labels. This outcome highlights the ability of the ACC data to capture fundamental biomechanical patterns that characterize different types of activity. The clustering approach carried out, through its staged refinement, identified six meaningful clusters that align closely with natural clusterings in the data. The NMI (0.728), accuracy (81.7%), and F1 score (81.2%) support the high quality of this clustering, while SC (0.314) indicates reasonable cluster cohesion given the complexity of human motion data.

In contrast, ECG-based recognition was driven more strongly by differences between subjects than by activity type. The dissimilarity matrix and embedding revealed horizontal and vertical line patterns indicative of consistent cardiac signatures unique to individual participants. Although some degree of grouping by subject was observed, overlap between trials in the embedded space led to ambiguities and less accurate clustering. This led to moderate clustering results for subject recognition, with an NMI of 0.641, accuracy around 50.0%, and a F1 score near 53.0%. The SC of 0.261 suggests limited cluster compactness, reflecting physiological variability and some overlap in cardiac patterns across subjects.

When combining ECG and ACC signals, the clustering result showed increased complexity. The dissimilarity structure and the spatial embedding suggested that the formed clusters were influenced simultaneously by activity and identity factors, but did not align clearly with either. The combined modality, while richer in information, introduced overlapping patterns that reduced the separability of clusters. As a result, performance decreased for both activity recognition and subject identification, with accuracy decreasing to 55.0% and 45.0%, respectively, and corresponding decreases in F1 score and precision. The SC also decreased, showing less cohesive clusters.

**Figure 20:**
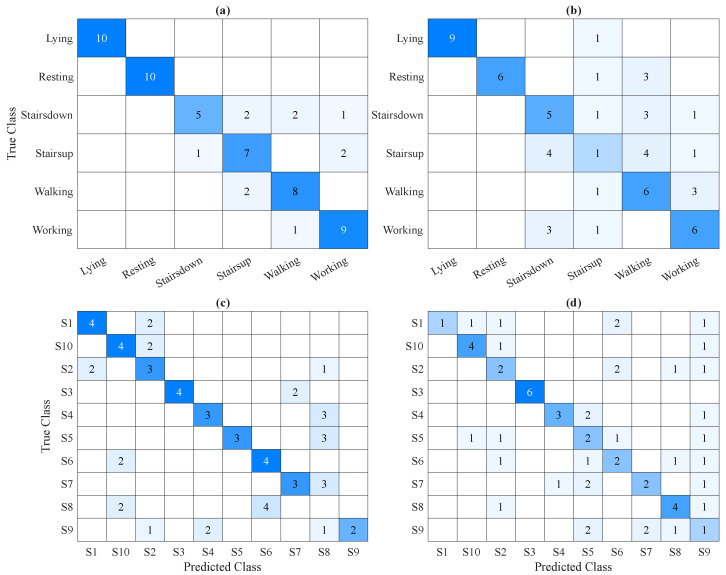
Confusion matrices illustrating classification performance. (**a**) Activity recognition using ACC signals. (**b**) Activity recognition using combined ACC + ECG signals. (**c**) Subject identification using ECG signals. (**d**) Subject identification using combined ACC + ECG signals. The intensity of blue color represents the value levels.

The confusion matrices (Figure 20) confirm these trends, showing more misclassifications and less distinct cluster boundaries in the combined modality compared to ACC or ECG alone. This suggests that, without careful feature integration or dimensionality reduction, merging heterogeneous data sources encoding different types of information can introduce confounding factors that obscure the relevant patterns for either task.

In the context of activity recognition based solely on the ACC sensor, lying and resting are recognized with high accuracy, due to distinct acceleration values, particularly along the Y and Z axes. These differences make it easy for the model to separate static postures. However, within more dynamic activities, misclassifications do occur, especially with climbing stairs, which is often confused with descending stairs and working. This is likely due to the similar movement patterns and intensity levels of these activities. When the ECG sensor is added to the system, the overall recognition performance deteriorates. In particular, the classification of climbing stairs becomes less accurate, as it is increasingly misidentified as other activities. Surprisingly, even the recognition of resting becomes less reliable, suggesting that the inclusion of ECG data may have introduced noise or confusion rather than improving the model’s ability to differentiate between activities.

For subject identification based on ECG signals, the results are more varied. Some individuals are occasionally misclassified, and one person received notably low accuracy scores, while others achieved similar and more consistent performance levels. When ACC data are added to the ECG signal, the recognition accuracy improves for three individuals, but, conversely, it worsens for several others. This suggests that the fusion of ECG and ACC data benefits some cases but introduces additional challenges or noise for others, leading to mixed overall results.

These findings highlight the value of treating each signal modality independently in unsupervised learning contexts. ACC signals exhibit clear biomechanical patterns that are suitable for activity discrimination, whereas ECG signals carry informative physiological signatures for individual recognition. Unlike supervised classification, which optimizes for specific tasks using labeled data, the unsupervised approach reveals the inherent structure of the data. The observed decline in clustering performance when combining modalities without tailored fusion strategies suggests that naive integration of heterogeneous sources can obscure rather than enhance the discovery of meaningful clusters. Future work should explore advanced multimodal fusion techniques or feature selection methods that preserve the complementary strengths of each modality while minimizing interference and redundancy.

Table 3 presents a comparison of our unsupervised method with selected approaches from the literature on two key tasks: activity recognition and human identification.

Our unsupervised method that uses only accelerometer data achieves competitive accuracy, demonstrating that it can effectively capture relevant activity patterns without relying on labeled data. However, when ACC is combined with ECG signals, the accuracy of our method decreases, which may be attributed to the increased complexity of multimodal data in an unsupervised learning setting. Despite this, the main advantage of our approach lies in its independence from annotated datasets, which makes it particularly useful in scenarios where labeled data are limited or unavailable. Similarly, while our method based on ECG or combined ACC and ECG signals shows lower accuracy compared to other methods, it still reveals the potential of unsupervised techniques to extract meaningful features for human identification without requiring ground-truth labels.

In general, our unsupervised method offers a valuable trade-off between accuracy, simplicity, and data requirements. It presents a promising alternative for real-world applications where collecting large amounts of labeled data is impractical or costly.

One limitation of this study is the relatively small sample size, which may limit the generalizability of the findings. Despite this, the results provide valuable insights into the application of unsupervised methods for activity recognition and human identification. Future work should focus on validating the proposed approach on larger and more diverse datasets to strengthen the conclusions and enhance the robustness of the methodology.

## 6. Conclusions

This work presented a fully unsupervised methodology for the analysis of multimodal physiological recordings related to human activities, acquired through wearable accelerometers and ECG sensors. The proposed pipeline integrates time-series alignment, similarity computation using DTW, graph-based spatial embedding, and iterative clustering, enabling the identification of latent structures in the data without the need for labeled training samples or task-specific supervision.

Three different signal modalities were independently evaluated: ACC, ECG, and their combination. The results demonstrated that the unsupervised approach was able to reveal meaningful patterns associated with both activity recognition and subject identification. Specifically, ACC signals provided the most discriminative information for behavioral segmentation (NMI = 0.728, accuracy = 0.817), while ECG signals were more informative for individual identification (NMI = 0.641, accuracy = 0.500). The combined modality did not yield improved results, suggesting that naively merging heterogeneous signals can introduce redundancy or conflicting information in the clustering process.

Cluster performance was consistent and, in some cases, comparable to classification-based methods reported in the literature, despite the absence of training labels or optimization for a specific recognition objective. These findings emphasize the potential of unsupervised learning strategies in exploratory analyses of physiological time series, particularly in scenarios where annotated data are limited or unavailable.

Future directions include extending the method to longer-duration recordings, incorporating additional sensing modalities (e.g., respiration, skin temperature), and exploring adaptive or online clustering frameworks.

## Figures and Tables

**Table 1:** Participant demographics.

Person	Gender [F/M]	Age [Years]		Person	Gender [F/M]	Age [Years]
1	M	22		16	F	45
2	M	23		17	M	16
3	M	61		18	F	25
4	F	54		19	F	25
5	F	70		20	F	25
6	F	63		21	F	24
7	M	70		22	M	19
8	M	81		23	M	74
9	F	76		24	F	73
10	F	72		25	M	57
11	M	76		26	F	67
12	F	41		27	M	73
13	F	25		28	F	24
14	F	27		29	F	60
15	F	51		30	F	27

**Table 2:** Evaluation of the followed methodology.

	Activity Recognition		Human Identification	
	ACC	ACC + ECG	ECG	ACC + ECG
**Clustering Metrics**				
NMI	0.728	0.473	0.641	0.474
SC	0.314	0.211	0.261	0.180
**Classification Metrics**				
Accuracy	0.817	0.550	0.500	0.450
F1 Score	0.812	0.552	0.530	0.458
Recall	0.817	0.550	0.500	0.450
Precision	0.824	0.584	0.624	0.539

**Table 3:** Comparison of methods for activity recognition and human identification.

Sensor	Study	Methods	Details	Accuracy
**Activity recognition**				
**ACC**	**Our study**	**Pairwise comparison**		**0.817**
		**+ 3D embedding + Clustering**		
ACC + Gyro	[18]	FA, MDS, PCA, SVD, SVM,	No data fusion	0.725
		LDA, DT, KNN		
			Decision-level data fusion	0.744
			Feature-level data fusion	0.674
			Sensor-level data fusion	0.593
ACC + Gyro	[7]	DL	Raw last layer	0.53–0.73
			Raw whole net	0.82–0.95
			Raw ACC + Gyro last layer	0.43–0.71
			Raw ACC + Gyro whole net	0.45–0.84
			HG last layer	0.76–0.82
			HG whole net	0.9–0.98
ACC	[15]	MARS		UAR = 0.831
**ACC + ECG**	**Our study**	**Pairwise comparison**		**0.550**
		**+ 3D embedding + Clustering**		
ACC + ECG	[24]	Bilateral GRU	Dataset I/Dataset II	0.991/0.971
ACC + ECG + Gyro	[23]	CNN	Intraperson classification	0.590–0.940
			Interperson classification	0.350–0.770
ACC+ECG	[25]	CNN-LSTM	Dataset I/Dataset II	0.984/0.942
**Human identification**				
**ECG**	**Our study**	**Pairwise comparison**		**0.5**
		**+ 3D embedding + Clustering**		
ECG	[6]	CSM		0.913
ECG	[5]	Distance-based metrics	Fiducial Points	0.944
ECG	[13]	1D-ResNet + SCA		0.991
**ACC + ECG**	**Our study**	**Pairwise comparison**		**0.45**
		**+ 3D embedding + Clustering**		
ACC + ECG + Gyro	[27]	CNN	Time-domain features	0.970
			Frequency-domain features	0.980
ACC + ECG + PPG + GSR	[26]	Gaussian Model		AUC = 0.996

## Data Availability

The data supporting the findings of this study are not publicly available due to privacy considerations.

## Abbreviations

The following abbreviations are used in this manuscript:

ACC	Accelerometer
AUC	Area Under the Curve
CNN	Convolutional Neural Network
CSM	Collaborative-Set Measurement
DL	Deep Learning
DT	Decision Tree Classifier
DTW	Dynamic Time Warping
ECG	Electrocardiogram
FA	Factor Analysis
GRU	Gated Recurrent Unit
GSR	Galvanic Skin Response
Gyro	Gyroscope
HAC	Hierarchical Agglomerative Clustering
HAR	Human Activity Recognition
HG	High Gain
IMU	Inertial Measurement Unit
KNN	K-Nearest Neighbors
LDA	Linear Discriminant Analysis
LSTM	Long Short-Term Memory
MARS	Multiview Contrastive Approach to HAR from ACC Sensor
MDS	Multidimensional Scaling
NMI	Normalized Mutual Information
PCA	Principal Component Analysis
PPG	Photoplethysmogram
ResNet	Residual Network
RNN	Recurrent Neural Network
SC	Silhouette Coefficient
SCA	Sequence Channel Attention
STFT	Short-Time Fourier Transform
SVD	Singular Value Decomposition
SVM	Support Vector Machine
UAR	Unweighted Average Recall
